# Association between High Serum Homocysteine Levels and Biochemical Characteristics in Women with Polycystic Ovarian Syndrome: A Systematic Review and Meta-Analysis

**DOI:** 10.1371/journal.pone.0157389

**Published:** 2016-06-09

**Authors:** Yuming Meng, Xiang Chen, Zheng Peng, Xuexiang Liu, Yifan Sun, Shengming Dai

**Affiliations:** 1 Department of Clinical Laboratory, the Fourth Affiliated Hospital of Guangxi Medical University, Liuzhou, Guangxi, China; 2 Department of Clinical Laboratory, Liuzhou Hospital of Traditional Chinese Medicine, Liuzhou, Guangxi, China; Zhejiang University, CHINA

## Abstract

**Background:**

Elevated homocysteine levels have been observed in previous studies of PCOS; however, the nature of the associations between high homocysteine levels and the biochemical characteristics of polycystic ovarian syndrome (PCOS)—such as obesity, insulin resistance (IR), and androgen levels—is still uncertain.

**Methods:**

A systematic search was conducted electronically up to December 28, 2015 using specific eligibility criteria. Standardized mean difference (SMD) and the corresponding 95% confidence intervals (95% CIs) were used as a measure of effect size.

**Results:**

A total of 34 studies (with 1,718 cases and 1,399 controls) of homocysteine levels in PCOS were pooled in this meta-analysis. Significantly lower homocysteine levels were found in controls than in PCOS patients (SMD = 0.895, 95% CI = 0.643–1.146, *P*<0.001; *I*^*2*^ = 90.4% and *P*<0.001 for heterogeneity), regardless of the degree of obesity, IR, or androgen levels. Homocysteine levels in non-IR PCOS patients were significantly lower than those of PCOS patients with IR (SMD = 0.69, 95% CI = 0.37–1.01, *P*<0.01; *I*^*2*^ = 0% and *P* = 0.50 for heterogeneity). However, metformin treatment did not appear to cause any significant change in the homocysteine levels of PCOS patients (SMD = –0.17, 95% CI = –1.10–0.75, *P* = 0.71; *I*^*2*^ = 92% and *P*<0.01 for heterogeneity).

**Conclusions:**

High homocysteine levels in women with PCOS are not related to degree of obesity, IR, or androgen levels. Metformin treatment cannot decrease the homocysteine levels in PCOS patients.

## Introduction

Polycystic ovarian syndrome (PCOS) is the most common endocrine abnormality, affecting 6–14% of reproductive-aged women [1]. However, PCOS is not a gynecological disorder alone, and obesity, insulin resistance (IR), and metabolic syndrome frequently appear in women with PCOS, which leads to increased incidence of longer-term health consequences, such as type 2 diabetes mellitus and cardiovascular events [2]. Therefore, the importance of managing PCOS in order to prevent the development of cardiovascular disease (CVD) has been recognized [3].

CVD has various risk factors, such as elevated blood pressure, cholesterol, or glucose levels and smoking. Homocysteine, a sulfur-containing amino acid, is associated with an increased risk of atherosclerotic and thromboembolic disorders as well as hyperinsulinemia [4]. Researchers have considered elevated homocysteine levels a “classical” risk factor for CVD [5]. Hyperhomocysteinemia may arise from nutritional deficiencies of folate, vitamin B_6_, and vitamin B_12_ [6]. Indeed, IR and obesity have been associated with lower serum vitamin B_12_ concentrations in PCOS patients [7]. Elevated serum total testosterone level, another biochemical characteristic of PCOS, is also a main factor associated with hyperhomocysteinemia in PCOS patients [8]. Previous meta-analyses have demonstrated higher homocysteine levels in PCOS patients than in healthy controls [9, 10]. Therefore, high circulating homocysteine levels may be an intrinsic characteristic of PCOS, and lowering homocysteine levels offers new possibilities for preventing CVD in women with PCOS.

Over the past decade, many studies have investigated homocysteine levels in women with PCOS; in addition, the relationships between homocysteine and PCOS’ biochemical characteristics—such as obesity, IR, and elevated androgen levels—have also been researched [7, 11–43]. However, these studies have shown inconsistent results, and the mechanism for high homocysteine levels in women with PCOS is still not fully understood. Although two previous meta-analyses have shown high homocysteine levels in PCOS patients, they did not illustrate the relationship between elevated homocysteine levels and biochemical characteristics of women with PCOS, which would limit the application of managing homocysteine levels in the treatment of PCOS. The purpose of this systematic review and meta-analysis is to investigate the relationship between characteristics of PCOS and homocysteine levels. In addition, metformin is widely used in PCOS patients for IR treatment. Vitamin B12 level is known to decrease during metformin treatment [44], and vitamin B12 is strongly associated with serum homocysteine [45]. Hence, homocysteine level might increase during metformin treatment. We therefore further investigate the effect of metformin treatment on homocysteine levels in PCOS patients.

## Materials and Methods

The present meta-analysis was performed according to the PRISMA statement (Preferred Reporting Items for Systematic Reviews and Meta-Analyses) [46] (S1 Table). There was no registered protocol.

### Identification of studies and eligibility criteria

Two investigators (YM and XC) independently conducted a systematic literature search in PubMed, Embase, and the Cochrane Library database for relevant studies from inception through December 2015. In addition, we searched the reference lists of all identified relevant publications, review and meta-analysis articles. Only English language and human studies were searched. Disagreement was resolved by group consultation.

The literature search using exploded Medical Subject Headings (MeSH) terms and corresponding keywords. The search terms used were: (MeSH exp ‘Polycystic Ovary Syndrome’ and keywords ‘PCOS’), and (MeSH exp ‘Homocysteine’ and keywords ‘Hcy’).

For the study selection, the first eligibility criteria was PCOS clearly defined according to the Rotterdam criteria [47] or National Institute of Health (NIH) criteria [48] or Androgen Excess & PCOS Society 2006 criteria[49]. The second criteria was that body mass index (BMI) and age should be matched between PCOS patients and controls. All included studies perfectly meet the above two criteria and also include serum homocysteine means and standard deviations (SDs) for cases and controls. To best understand the association between homocysteine and IR in PCOS, studies of homocysteine levels in PCOS patients treated with metformin were also included in this meta-analysis.

Studies without a control group or subjects with diseases other than PCOS were excluded. Letters, case reports, editorials, and conference abstracts were also excluded.

### Quality score assessment

The quality of the included studies in this meta-analysis was assessed according to the Newcastle-Ottawa Quality Assessment Scale (NOS) [50], and the assessment items were established according to the study by Murri et al. [10]. Studies with an NOS score ≤5 were considered “medium” quality studies. “High” quality studies were defined as having a quality score >5. The maximum NOS score was 8.

### Data extraction

Two investigators (YM and XC) independently extracted the following data from the retrieved studies: name of the first author, publication date, country, sample size, diagnostic criteria for PCOS, matched factors, mean BMI and age, mean serum homocysteine level and standard deviation (SD), measurement method, IR status, and androgen status. The two researchers attempted to obtain the necessary data by contacting the authors of the original articles as needed (i.e., when the homocysteine levels were reported as median or geometric values). The data were extracted by the two investigators were expected to be the same, and any dispute was resolved by group consultation.

### Statistical analysis

Homocysteine levels were extracted as the mean ± SD from the included studies. The assessment of IR was based on the ratio of the mean Homeostasis Model Assessment of Insulin Resistance (HOMA-IR) values in the PCOS group to that of the controls (HOMA-IR ratio). Similarly, androgen status was assessed according to the ratio of the mean testosterone level (total or free) in PCOS to that of the controls (T ratio). Because various measurement methods and units for homocysteine level were used across studies, we used standardized mean difference (SMD) but not weighted mean difference (WMD), with the corresponding 95% confidence intervals (95% CIs) as a measure of effect size in this meta-analysis.

Heterogeneity was assessed using a chi-squared Q test and I-squared statistics. If *P*_*Q*_<0.1 or *I*^*2*^>50%, the heterogeneity was considered significant, and a random-effects model (the DerSimonian and Laird method) was used. Otherwise, a fixed-effects model was used. To better understand the relationship between homocysteine level and the basic characteristics of PCOS, such as BMI, IR status, and androgen status, the corresponding subgroup analysis was carried out according to predefined criteria. BMI values were used to categorize patients into a normal-weight group (BMI <25 kg/m^2^) or an over-weight group (BMI ≥25 kg/m^2^). Quartile intervals were used to categorize patients according to HOMA2-IR ratio and T ratio.

If statistically significant heterogeneity was observed in this meta-analysis, a univariate meta-regression analysis for the potential variables between studies was first performed. In the meta-regression model, the SMD was used as the dependent variable, and potential confounder factors—such as the diagnosis criteria, BMI, NOS score, HOMA-IR ratio, and T ratio—were used as covariates. Covariates with values of *P*>0.05 were not considered to be sources of heterogeneity. If the variables were significant at the 0.1 level, a multivariable analysis was further performed.

Sensitivity analysis was used to examine the influence of individual studies by excluding the studies one by one [51]. Publication bias was estimated by Begg’s test and further confirmed by Egger’s regression [51].

RevMan 5.2.7 (Cochrane Collaboration) and STATA software (version 12.0; Stata Corporation, College Station, TX) were used in this meta-analysis.

## Results

### Literature selection

**Fig 1:**
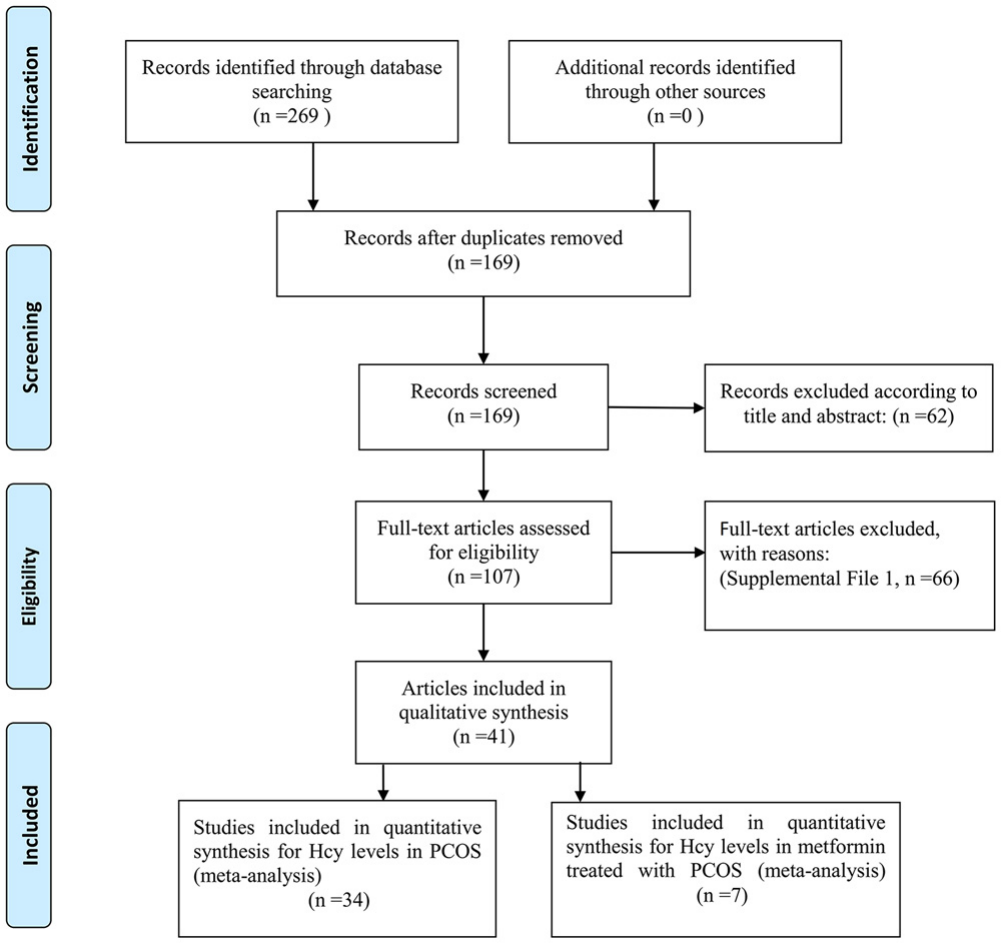
Flow diagram of included studies for this meta-analysis.

This meta-analysis finally included 41 eligible studies, of which 34 [7, 11–43] were in quantitative synthesis for homocysteine levels in PCOS, and seven [21, 22, 52–56] covered homocysteine levels in PCOS patients treated with metformin. Fig 1 shows the search flow diagram; in addition, the excluded articles with reasons for exclusion were shown in S1 File.

### Characteristics of the included studies

For the serum homocysteine level in women with PCOS, the characteristics of the included studies are shown in Table 1. All the included studies were age and BMI matched between PCOS and control groups except the study by Yarali et al. [18]. In addition, five studies further matched PCOS and control groups in terms of B_12_ and folate levels [7, 14, 22, 25, 35]. The selection of PCOS patients in seven studies [15, 16, 18, 26, 35, 36, 42] was according to NIH, and the other 27 studies [7, 11–14, 17, 19–25, 27–34, 37–41, 43] used the Rotterdam criteria. Three studies were assessed as “medium” quality [18, 35, 43] and the other 31 were all “high” quality studies according to NOS. For studies covering homocysteine levels in PCOS patients treated with metformin, the metformin dose and duration of treatment are shown in Table 2, as well as the other basic characteristics of PCOS patients, such as the definition of PCOS, BMI, sample size, and homocysteine levels before and after treatment.

**Table 1:** Characteristics of included studies in meta-analysis.

Study	Year	Country	Detecting method	Diagnosis criteria	Matched factors	BMI	No. _PCOS/Control_	HOMA ratio	T ratio	NOS score
Yarali	2001	Turkey	FPIA	NIH	BMI,B12,Folate	≥25	30/30	NR	1.42	5
Orio	2003	Italy	HPLC	NIH	Age,BMI,B12,Folate	<25	70/70	3.30	1.65	6
Kilic-Okman	2004	Turkey	Chemiluminescence	NIH	Age, BMI	≥25	29/25	1.82	NR	7
Bayraktar	2004	Turkey	HPLC	NIH	Age,BMI,B12,Folate	<25	50/25	1.62	1.80	4
Bickerton	2005	United Kingdom	ETMS	NIH	Age, BMI	≥25	11/12	NR	1.56	6
Yilmaz	2005	Turkey	HPLC	ESHRE/ASRM	Age,BMI,B12,Folate	<25	50/35	1.46	1.46	6
Topcu	2006	Turkey	NR	ESHRE/ASRM	Age, BMI	≥25	28/26	1.67	1.83	6
Sahin	2007	Turkey	FPIA	ESHRE/ASRM	Age, BMI	<25	20/20	NR	3.16	7
Guzelmeric	2007	Turkey	FPIA	NIH	Age, BMI	≥25	44/26	1.61	1.88	7
Atamer	2008	Turkey	Fluorescence detection	ESHRE/ASRM	Age, BMI	≥25	35/30	1.72	2.31	6
Heutling	2008	Germany	HPLC	ESHRE/ASRM	Age, BMI	≥25	83/39	1.72	1.67	8
Battaglia	2008	Italy	FPIA	NIH	Age, BMI	≥25	28/15	2.07	2.13	7
Kaya	2009	Turkey	HPLC	ESHRE/ASRM	Age,BMI,B12,Folate	≥25	61/61	2.06	1.75	8
Oktem	2009	Turkey	FPIA	ESHRE/ASRM	Age, BMI	≥25	31/31	1.63	1.40	5
Cetinkalp	2009	Turkey	NR	ESHRE/ASRM	Age, BMI	<25	129/91	2.68	0.93	7
Soares	2009	Brazil	Chemiluminescence	ESHRE/ASRM	Age, BMI	<25	40/50	1.04	1.54	7
Kaya	2010	Turkey	HPLC	ESHRE/ASRM	Age, BMI	<25	68/68	2.85	2.34	8
Nafiye	2010	Turkey	Chemiluminescence	ESHRE/ASRM	Age, BMI	≥25	36/61	1.92	NR	8
Karaer	2010	Turkey	Competitive immunoassay	ESHRE/ASRM	Age,BMI,B12,Folate	≥25	31/31	1.86	1.38	8
Fulghesu	2010	Italy	Chemiluminescence	ESHRE/ASRM	Age, BMI	<25	71/94	1.06	2.00	7
Karadeniz	2010	Turkey	FPIA	ESHRE/ASRM	Age, BMI	<25	86/70	2.64	34.33	6
Arikan	2010	Turkey	Chemiluminescence	ESHRE/ASRM	Age, BMI	<25	31/25	1.73	3.00	6
Mohamadin	2010	Saudi Arabia	ELISA	ESHRE/ASRM	Age, BMI	≥25	50/40	1.89	2.32	6
Temel	2010	Turkey	ELISA	ESHRE/ASRM	Age, BMI	<25	30/30	1.82	1.37	6
Salehpour	2011	Iran	RIA	ESHRE/ASRM	Age, BMI	≥25	85/83	NR	NR	6
Altug	2011	Turkey	FPIA	ESHRE/ASRM	Age,BMI,B12,Folate	<25	24/20	1.76	1.74	6
Hemati	2011	Iran	ELISA	ESHRE/ASRM	Age, physical activity	<25	64/50	2.52	2.21	7
Caglar	2011	Turkey	NR	ESHRE/ASRM	Age, BMI	<25	61/21	1.75	1.56	8
Bayrak	2012	Turkey	FPIA	ESHRE/ASRM	Age,BMI,smoking	<25	77/25	1.58	1.83	7
Deveer	2012	Turkey	Chemiluminescence	ESHRE/ASRM	Age, BMI	<25	25/25	1.74	1.28	8
Harmanci	2013	Turkey	HPLC	ESHRE/ASRM	Age, BMI	<25	23/23	1.28	1.96	7
Maleedhu	2014	India	EIA	ESHRE/ASRM	Age, BMI	<25	142/65	NR	NR	6
Moti	2015	Iran	HPLC	ESHRE/ASRM	Age, BMI	<25	30/30	1.82	NR	7
Miranda-Furtado	2015	Brazil	NR	ESHRE/ASRM	Age, BMI	≥25	45/52	1.80	1.21	7

FPIA, fluorescence polarization immunoassay; EIA, Enzyme Immunoassay; ETMS, electrospray tandem mass spectrometry; NIH, National Institute of Health criteria; ESHRE/ASRM, Rotterdam criteria; BMI, body mass index; NR, no reporting; NOS, Newcastle-Ottawa Quality Assessment Scale

**Table 2:** Characteristics of included studies for Hcy levels in metformin treated with PCOS.

Study	Year	Country	Definition of PCOS	Dose of treatment	Time of treatment	BMI	N_PCOS_	Before treatment		After treatment	
								Mean (μmol/l)	SD	Mean (μmol/l)	SD
Vrbikova	2002	Czech Republic	NIH	first week:500mg q.d., then 1000mg q.d.	7 months	≥25	9	10.1	2.6	13.4	5.1
Kilicdag	2005	Turkey	ESHRE/ASRM	850mg b.i.d.	3 months	≥25	14	9.56	0.81	11.97	1.01
Yilmaz	2005	Turkey	ESHRE/ASRM	850 mg b.i.d.	3 months	<25	25	13.52	5.98	13.46	6.01
Sahin	2007	Norway	ESHRE/ASRM	2550 mg q.d.	6 months	<25	20	12.8	5.2	12.3	5.6
Schachter	2007	Israel	ESHRE/ASRM	1700mg q.d.	3 months	≥25	28	10.5	0.9	8.6	0.7
Haydardedeoglu	2009	Turkey	ESHRE/ASRM	850 mg b.i.d.	3 months	≥25	20	8.74	1.9	10.97	3.31
Rajagopal	2012	India	NIH	first week: 500mg q.d. next week: 500mg b.i.d. then to 1,000mg b.i.d.	3 months	≥25	25	20	7.3	18.2	8.9

NIH, National Institute of Health criteria; ESHRE/ASRM, Rotterdam criteria; BMI, body mass index; SD, standard deviations

### Meta-analysis of homocysteine levels in women with PCOS

**Fig 2:**
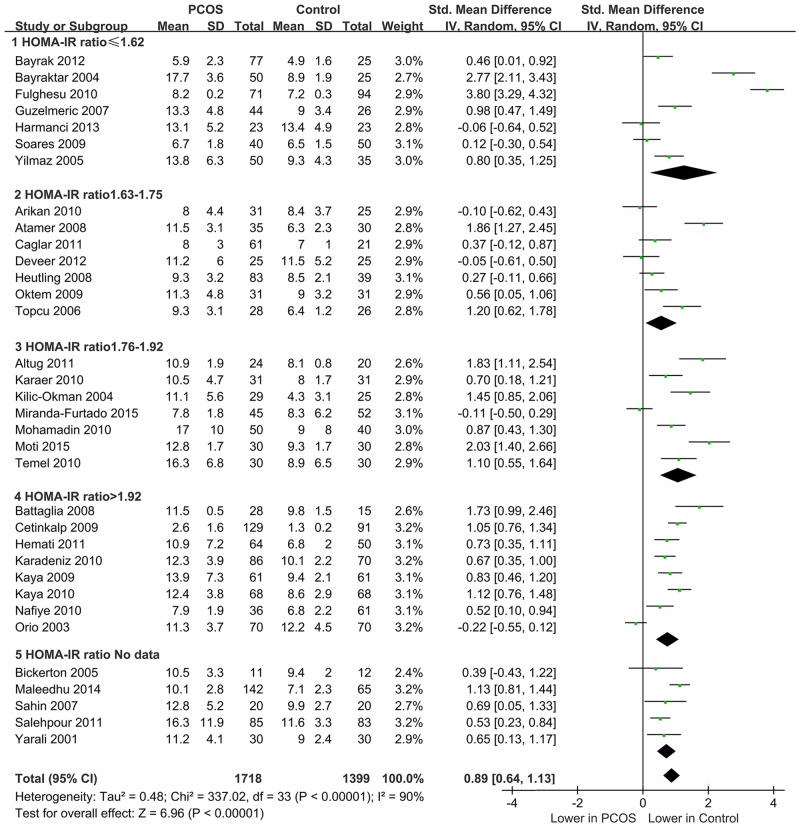
Forest plot of the pooled quantitative synthesis and subgroup analysis stratified by HOMA-IR ratio.

When the 34 included studies with 1,718 cases and 1,399 controls were pooled in meta-analysis, significantly lower homocysteine levels were found in controls than in PCOS patients (SMD = 0.895, 95% CI = 0.643–1.146, and *P*<0.001). However, significant heterogeneity was observed across studies (*I*^*2*^ = 90.4% and *P*<0.001, Fig 2 and Table 3).

**Table 3:** Subgroup analysis results in meta-analysis.

Subgroup	No. study	No. PCOS	No. Control	SMD (95%CI)	*P*_value_	Heterogeneity	
						*I*^*2*^%	*P*_value_
**Overall**	34	1718	1399	0.895(0.643–1.146)	<0.001	90.4	<0.001
**Diagnosis criteria**							
NIH	7	262	203	1.109(0.324–1.895)	0.006	92.9	<0.001
ESHRE/ASRM	27	1456	1196	0.845(0.579–1.111)	<0.001	90.0	<0.001
**BMI**							
Over-weight	14	597	532	0.817(0.547–1.088)	<0.001	77.6	<0.001
Normal-weight	20	1121	867	0.940(0.557–1.323)	<0.001	93.3	<0.001
**HOMA-IR ratio**							
≤1.62	7	355	278	1.271(0.241–2.301)	0.016	96.6	<0.001
1.63–1.75	7	294	197	0.582(0.099–1.065)	0.018	84.0	<0.001
1.76–1.92	7	239	228	1.110(0.538–1.681)	<0.001	87.7	<0.001
>1.92	8	542	486	0.776(0.424–1.128)	<0.001	85.9	<0.001
No data	5	288	210	0.738(0.439–1.037)	<0.001	51.7	0.082
**T-ratio**							
<1.54	8	371	325	0.598(0.260–0.937)	0.001	77.5	<0.001
1.54–1.79	7	350	273	0.479(0.052–0.906)	0.028	83.5	<0.001
1.80–2.20	7	321	234	1.567(0.519–2.614)	0.003	95.9	<0.001
>2.20	7	354	303	0.836(0.474–1.197)	<0.001	78.6	<0.001
NR	5	322	264	1.095(0.607–1.583)	<0.001	85.1	<0.001
**NOS score**							
Medium	3	188	146	1.040(0.664–1.416)	<0.001	51.0	0.130
High	31	1530	1253	0.881(0.605–1.156)	<0.001	91.0	<0.001

NIH, National Institute of Health criteria; ESHRE/ASRM, Rotterdam criteria; BMI, body mass index; HOMA-IR, Homeostasis Model Assessment of Insulin Resistance; T, testosterone; NOS, Newcastle-Ottawa Quality Assessment Scale

To investigate the association between characteristics of PCOS patients and homocysteine level, subgroup analyses were performed. Studies with data not reported or not available were categorized into a non-reporting (NR) group. Quartile intervals for HOMA2-IR ratios were ≤1.62, 1.63–1.75, 1.76–1.92, and >1.92. Quartile intervals for T ratios were <1.54, 1.54–1.79, 1.80–2.20, and >2.20. As shown in Table 3, homocysteine levels in controls were significantly lower than those of PCOS patients in all predefined categories for HOMA2-IR ratio (Fig 2) and T ratio (S1 Fig). Similar results were found in the subgroup analyses according to diagnosis of PCOS, BMI (S2 Fig), and NOS score. However, significant heterogeneity was still found in all subgroup analyses. Meta-regression analyses were carried out to further investigate the source of heterogeneity. However, none of the potential variables between studies—such as quality score, BMI, diagnosis criteria, HOMA2-IR ratio, and T ratio—contributed to heterogeneity (*P*>0.05, Table 4).

**Table 4:** Univariate meta-regression analysis for the potential variables between studies.

Covariates	No. study	Coefficient	SD	*t*	*P*	95%CI
Quality Score	34	-0.172	0.517	-0.33	0.741	[-1.227, 0.881]
BMI	34	-0.098	0.3	-0.33	0.743	[-0.709, 0.511]
HMOA-IR ratio	29	-0.38	0.335	-1.13	0.268	[-1.068, 0.309]
T ratio	29	-0.004	0.027	-0.16	0.876	[-0.060, 0.052]
Diagnosis Criteria	34	-0.252	0.368	-0.69	0.497	[-1.002, 0.496]

BMI, body mass index; HMOA-IR, Homeostasis Model Assessment of Insulin Resistance; T, total testosterone; SD, standard error

**Fig 3:**
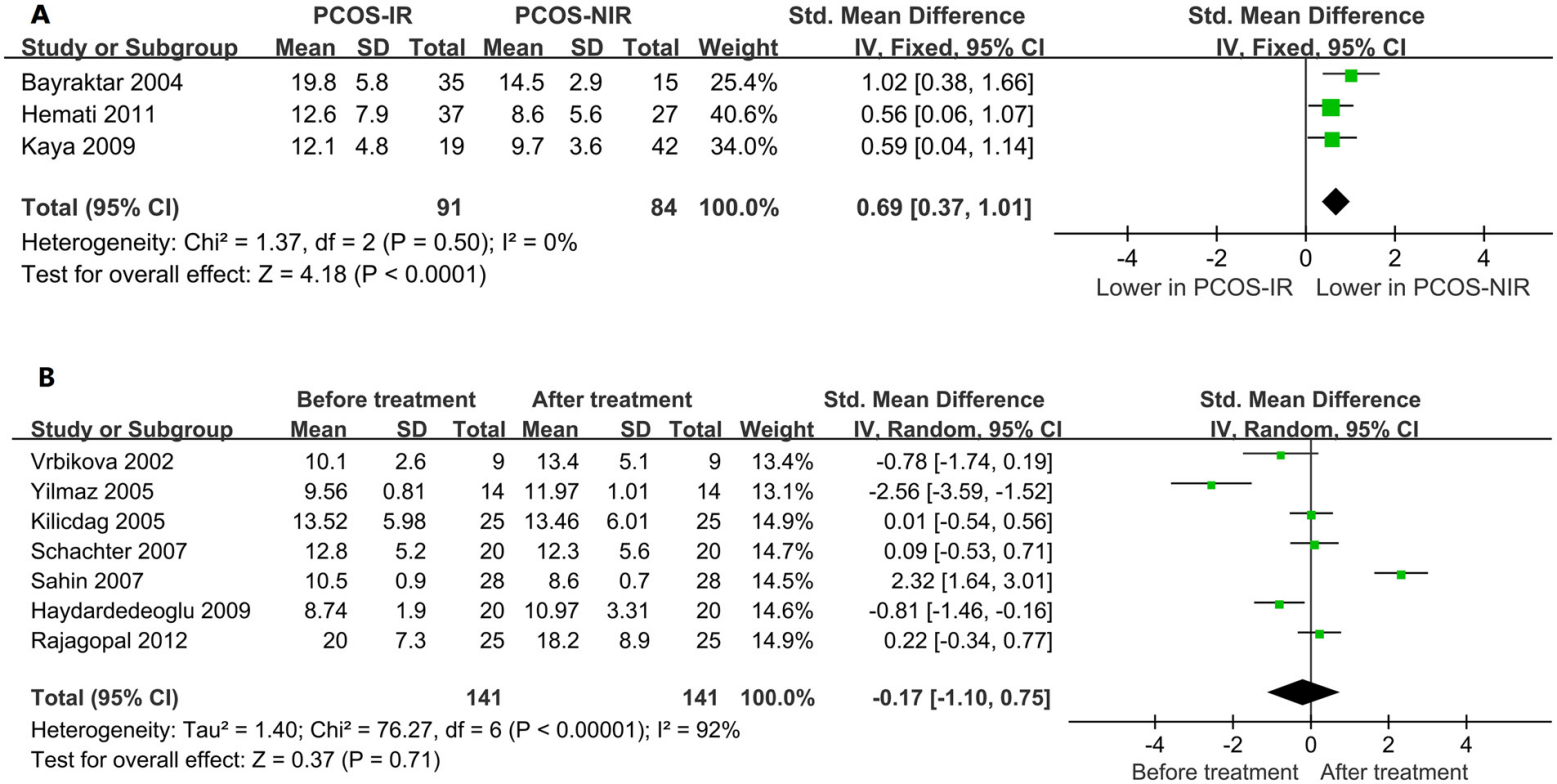
Forest plot of the influence of IR on homocysteine levels. A: PCOS patients with IR were compared with non-IR patients. B: Influence of metformin treatment on homocysteine levels.

To investigate the influence of IR on homocysteine levels in women with PCOS, PCOS patients with IR were compared with non-IR patients. As shown in Fig 3A, the homocysteine levels in non-IR PCOS patients were significantly lower than those of PCOS patients with IR (SMD = 0.69, 95% CI = 0.37–1.01, *P*<0.01; *I*^*2*^ = 0% and *P* = 0.50 for heterogeneity). However, metformin treatment did not appear to cause any significant change in the homocysteine levels of PCOS patients (SMD = –0.17, 95% CI = –1.10–0.75, *P* = 0.71; *I*^*2*^ = 92% and *P*<0.01 for heterogeneity, Fig 3B).

### Sensitivity analysis and publication bias

The pooled SMDs and 95% CIs were not significantly changed when each study was omitted (S3 Fig). The study by Fulghesu et al. [31] is of particular interest; it showed an impressive positive result, but the pooled SMDs and 95% CIs were not significantly changed when it was excluded (SMD = 0.79, 95% CI = 0.59–0.99, and *P*<0.001).

The results from Begg’s test showed no significant publication bias in this meta-analysis, and Egger’s regression results further confirmed this point (t = 1.01, *P*_*Egger’s*_ = 0.32, and 95% CI = –0.50–1.49).

## Discussion

In the present systematic review and meta-analysis, our results demonstrated significantly elevated homocysteine levels in PCOS patients when compared with controls, regardless of the degree of obesity, IR, or androgen levels, yet with significant between-study heterogeneity. Moreover, our results indicate that metformin treatment did not influence the homocysteine levels in PCOS patients.

For the overall analysis, our results are consistent with previous meta-analyses performed by Toulis et al. [9] and Murri et al.[10], although there are many differences between these studies (such as the number of included studies, exclusion criteria, and meta-analysis techniques). In addition, our study further investigated the relationship between elevated homocysteine levels and BMI, IR status, and androgen status. Interestingly, hyperhomocysteinemia was found both in normal-weight and over-weight women with PCOS, suggesting that increased homocysteine levels were not related to obesity. Previous case-control studies had found that elevated plasma homocysteine levels in PCOS patients were independent from obesity [57]. Indeed, even in the absence of obesity, patients with PCOS frequently have excessive body fat and central adiposity [58]. Combined with the results from the meta-regression model, our results suggest that obesity may be not a confounding factor that must be adjusted for in studies of homocysteine levels in women with PCOS.

Most of the included studies have shown higher IR and testosterone levels in women with PCOS than in controls (HOMA-IR ratio and T ratio>1.0). Results from subgroup analysis indicate that homocysteine levels are elevated in PCOS patients regardless of the degree of IR and androgen levels. In addition, pooled results from three studies indicate that homocysteine levels were higher in PCOS patients with IR than in PCOS patients without IR. However, we did not find that homocysteine levels decreased after metformin treatment. These results suggest that IR may be one, but not the sole reason for the elevated homocysteine levels in women with PCOS. Indeed, an abnormal homocysteine status is not necessarily caused by a single factor; it is often the result of combined effects, such as age, gender, nutrition, and smoking status [59] as well as gene variants [60]. Hence, in one respect, our results suggest that metformin treatment may not decrease the risk of CVD by reducing homocysteine levels; however, combined with folate or vitamin B_12_ supplementation, it may be a beneficial additional treatment. In the meantime, our finding of no adverse effect of metformin on homocysteine homeostasis in PCOS women is reassuring. After all, metformin is the first-line drug in the treatment of PCOS.

Some limitations should be considered when examining the results of this meta-analysis. First, we found significant heterogeneity across eligible studies, which may reduce the reliability of our findings. We assessed heterogeneity using various statistical methods; however, the potential variables between studies—such as quality score, BMI, diagnosis criteria, HOMA2-IR ratio, and T ratio—were all not the main source of heterogeneity. Indeed, PCOS is a form of heterogeneous endocrinopathy with various clinical features, including hirsutism, acne, infertility, amenorrhea, and oligomenorrhea [2]. Moreover, most of the included studies in this meta-analysis have small sample sizes. In addition, the measurement for homocysteine levels in serum is not standardized, and various assays were used in previous studies. Considering these influences, it is understandable that significant heterogeneity was observed in our meta-analysis. Second, potential publication bias should be considered, despite the fact that Begg’s test and Egger’s regression showed no significant publication bias in this meta-analysis.

Despite these limitations, the strengths of our meta-analysis include a unique evaluation of the influence of the relationship between the main features of PCOS and homocysteine levels, especially in terms of obesity and IR status. Our results may shed some light on this unresolved issue. We also first evaluated the effect of metformin on homocysteine levels in PCOS patients. These are the main differences of this study compared with previously published systematic reviews and meta-analysis. In addition, the large number of subjects included in the analyses strengthen their statistical power, and we also strictly assessed literature quality according to NOS criteria. Finally, results from sensitivity analysis indicated that our conclusion was relatively stable.

## Conclusions

In conclusion, the present meta-analysis suggests that high homocysteine levels in women with PCOS are not related to degree of obesity, IR status, or androgen levels. Metformin treatment cannot decrease the homocysteine levels in PCOS patients.

## Supporting Information

S1 Fig Forest plot for IL-6 levels in PCOS patients compared with controls stratified by testosterone ratio. (TIF)

S2 Fig Forest plot for IL-6 levels in PCOS patients compared with controls stratified by BMI. (TIF)

S3 Fig Cumulative meta-analysis. (TIF)

S1 File The excluded studies with reasons. (PDF)

S1 Table PRISMA 2009 Checklist. (DOC)
