# Supplementary figures and images for: Association between High Serum Homocysteine Levels and Biochemical Characteristics in Women with Polycystic Ovarian Syndrome: A Systematic Review and Meta-Analysis
Source: PLoS One. 2016 Jun 9;11(6):e0157389. doi: 10.1371/journal.pone.0157389 (PMC4900592; doi:10.1371/journal.pone.0157389)

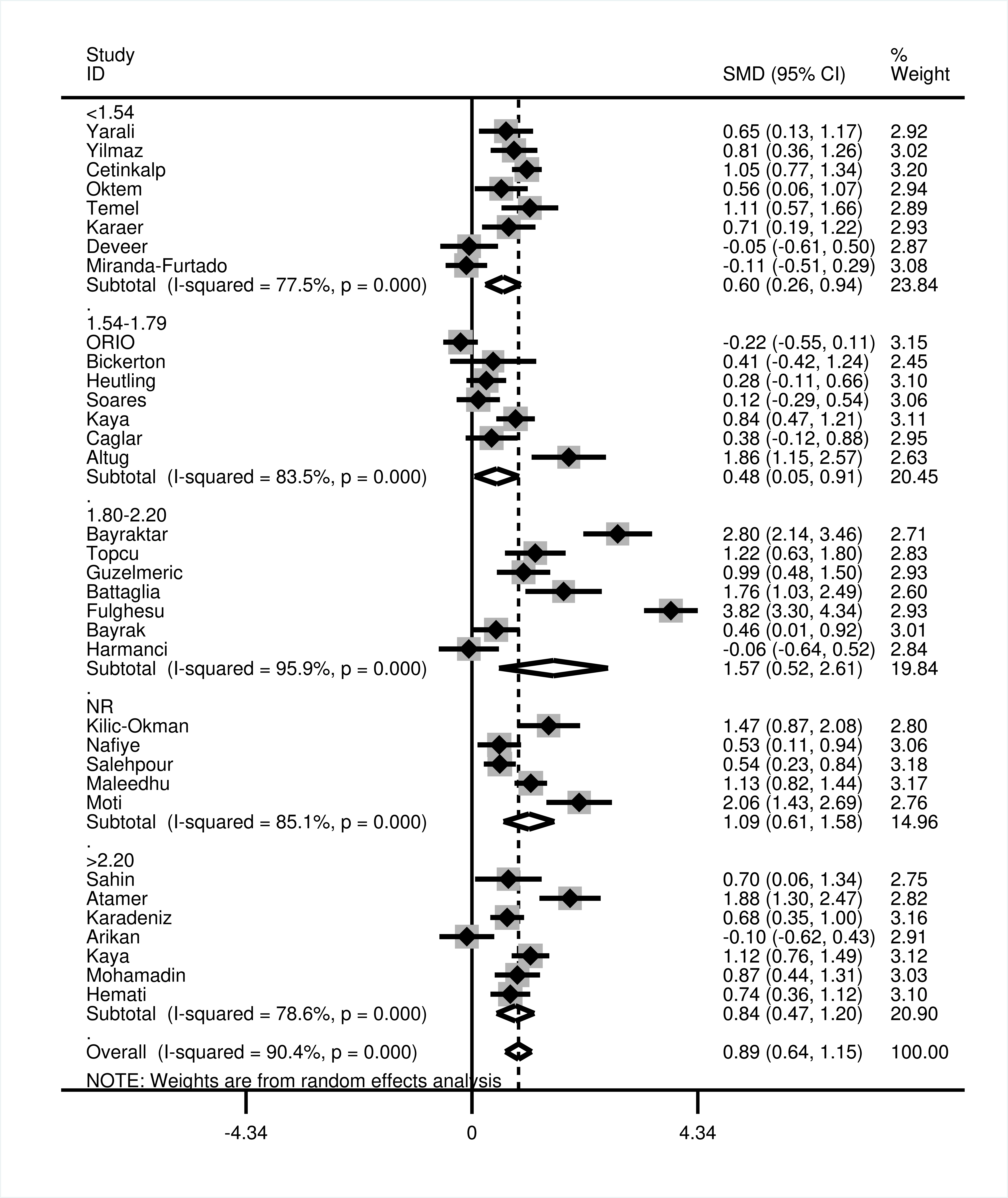

Supplement: S1 Fig — (TIF) [file pone.0157389.s001.tif]

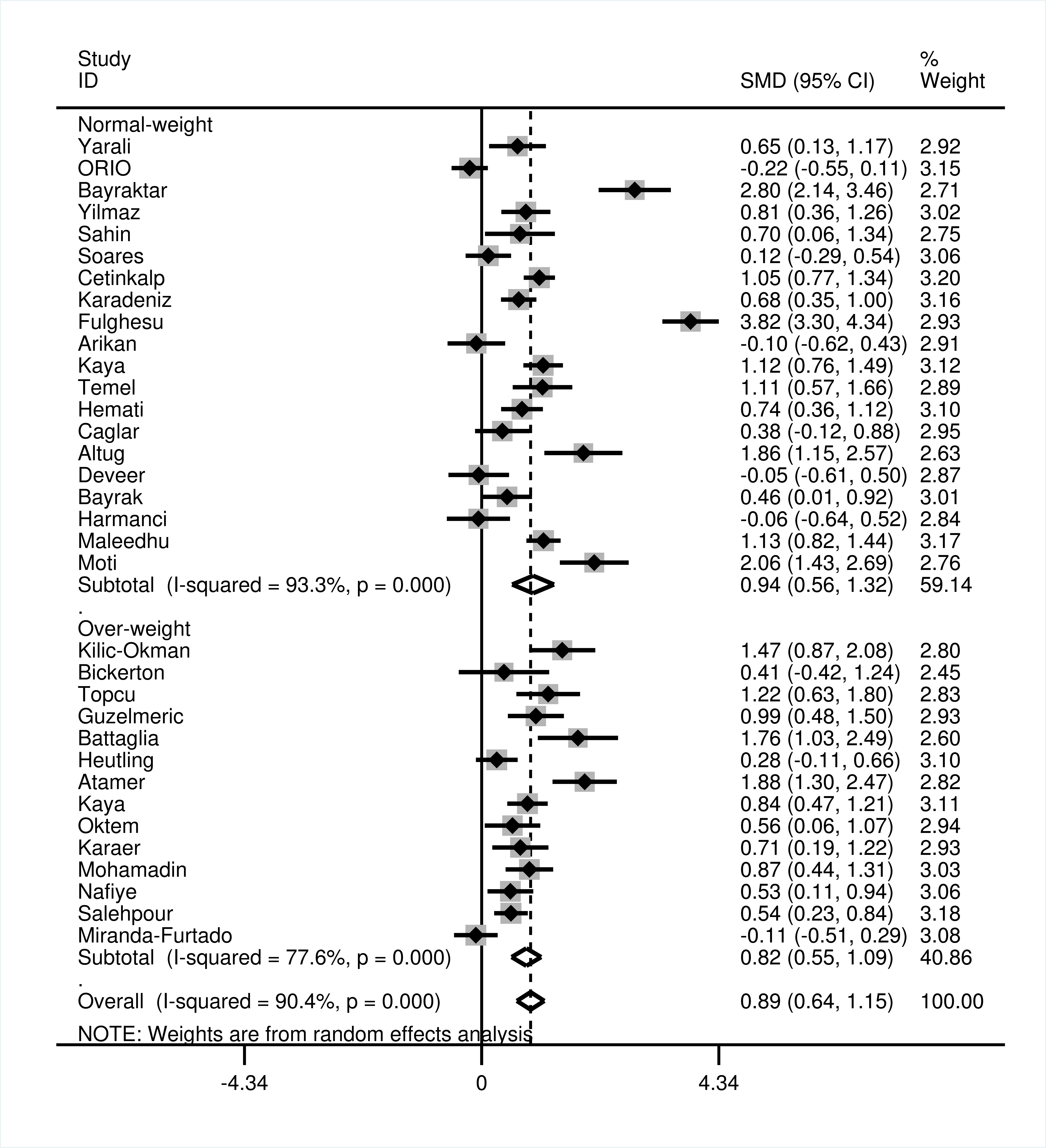

Supplement: S2 Fig — (TIF) [file pone.0157389.s002.tif]

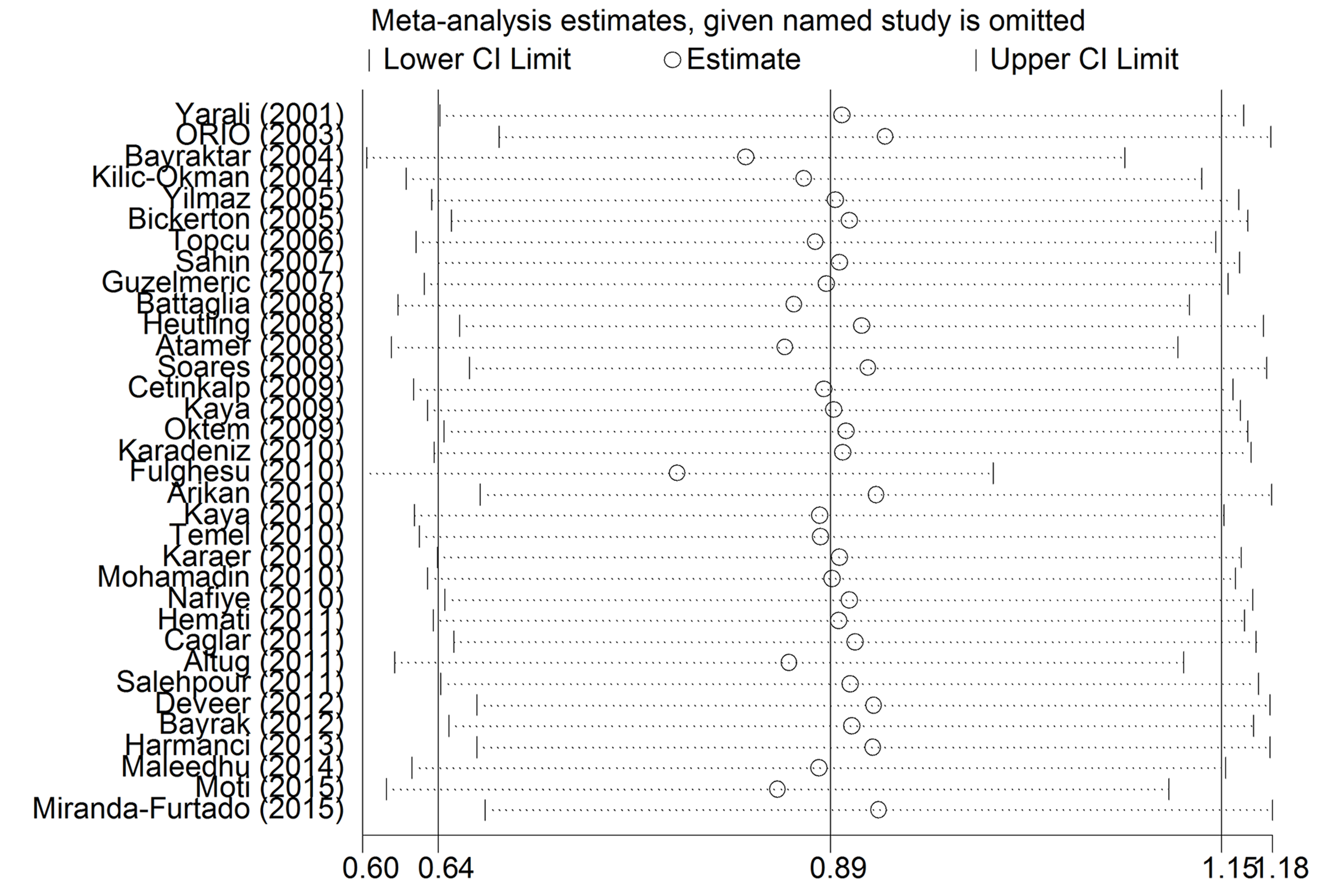

Supplement: S3 Fig — (TIF) [file pone.0157389.s003.tif]
