# Supplementary material for: Association between High Serum Homocysteine Levels and Biochemical Characteristics in Women with Polycystic Ovarian Syndrome: A Systematic Review and Meta-Analysis
Source: PLoS One. 2016 Jun 9;11(6):e0157389. doi: 10.1371/journal.pone.0157389 (PMC4900592; doi:10.1371/journal.pone.0157389)
Supplement: S1 File — (PDF) [file pone.0157389.s004.pdf]

Excluded with reasons:

No controls:[1-13]

Non-strict diagnosis of PCOS:[14]

Congress abstracts:[15-19]

No data:[20-25]

BMI not matched:[26-40]

Over-representation of cases by the same authors:[35, 41-49]

No response to requests for data clarification:[50]

Age not matched [51]

Other diseases[52]

1. Randeva HS, Lewandowski KC, Drzewoski J, Brooke-Wavell K, O'Callaghan C, Czupryniak L, et al. Exercise decreases plasma total homocysteine in overweight young women with polycystic ovary syndrome. The Journal of clinical endocrinology and metabolism. 2002;87(10):4496-501. Epub 2002/10/05. doi: 10.1210/jc.2001-012056. PubMed PMID: 12364425.
2. Kilicdag EB, Bagis T, Tarim E, Aslan E, Erkanli S, Simsek E, et al. Administration of B-group vitamins reduces circulating homocysteine in polycystic ovarian syndrome patients treated with metformin: a randomized trial. Human reproduction (Oxford, England). 2005;20(6):1521-8. Epub 2005/03/26. doi: 10.1093/humrep/deh825. PubMed PMID: 15790610.
3. Cagnacci A, Tirelli A, Renzi A, Paoletti AM, Volpe A. Effects of two different oral contraceptives on homocysteine metabolism in women with polycystic ovary syndrome. Contraception. 2006;73(4):348-51. Epub 2006/03/15. doi: 10.1016/j.contraception.2005.09.011. PubMed PMID: 16531164.
4. Gul OB, Somunkiran A, Yucel O, Demirci F, Ozdemir I. The effect of ethinyl estradiol-cyproterone acetate treatment on homocysteine levels in women with polycystic ovary syndrome. Archives of gynecology and obstetrics. 2008;277(1):25-30. Epub 2007/07/10. doi: 10.1007/s00404-007-0401-x. PubMed PMID: 17618446.
5. Makedos A, Goulis DG, Papanikolaou A, Panidis D. Serum high-sensitivity C-reactive protein and homocysteine changes during hormonal therapy in women with polycystic ovary syndrome: a prospective, matched study. Angiology. 2010;61(6):595-601. Epub 2010/04/17. doi: 10.1177/0003319709361198. PubMed PMID: 20395229.
6. Mancini F, Cianciosi A, Persico N, Facchinetti F, Busacchi P, Battaglia C. Drospirenone and cardiovascular risk in lean and obese polycystic ovary syndrome patients: a pilot study. American journal of obstetrics and gynecology. 2010;202(2):169.e1-8. Epub 2009/11/17. doi: 10.1016/j.ajog.2009.09.038. PubMed PMID: 19913778.

7. Kilicdag EB, Bagis T, Zeyneloglu HB, Tarim E, Aslan E, Haydardedeoglu B, et al. Homocysteine levels in women with polycystic ovary syndrome treated with metformin versus rosiglitazone: a randomized study. Human reproduction (Oxford, England). 2005;20(4):894-9. Epub 2004/12/25. doi: 10.1093/humrep/deh700. PubMed PMID: 15618250.
8. Schachter M, Raziel A, Strassburger D, Rotem C, Ron-El R, Friedler S. Prospective, randomized trial of metformin and vitamins for the reduction of plasma homocysteine in insulin-resistant polycystic ovary syndrome. Fertility and sterility. 2007;88(1):227-30. Epub 2007/03/06. doi: 10.1016/j.fertnstert.2006.11.071. PubMed PMID: 17336970.
9. Mancini F, Cianciosi A, Reggiani GM, Facchinetti F, Battaglia C, de Aloysio D. Endothelial function and its relationship to leptin, homocysteine, and insulin resistance in lean and overweight eumenorrheic women and PCOS patients: a pilot study. Fertility and sterility. 2009;91(6):2537-44. Epub 2008/05/09. doi: 10.1016/j.fertnstert.2008.03.023. PubMed PMID: 18462732.
10. Martins Wde P, Soares GM, Vieira CS, dos Reis RM, de Sa MF, Ferriani RA. [Cardiovascular risk markers in polycystic ovary syndrome in women with and without insulin resistance]. Revista brasileira de ginecologia e obstetricia : revista da Federacao Brasileira das Sociedades de Ginecologia e Obstetricia. 2009;31(3):111-6. Epub 2009/06/24. PubMed PMID: 19547885.
11. Kazerooni T, Ghaffarpasand F, Asadi N, Dehkhoda Z, Dehghankhalili M, Kazerooni Y. Correlation between thrombophilia and recurrent pregnancy loss in patients with polycystic ovary syndrome: a comparative study. Journal of the Chinese Medical Association : JCMA. 2013;76(5):282-8. Epub 2013/05/21. doi: 10.1016/j.jcma.2013.01.009. PubMed PMID: 23683262.
12. Morotti E, Battaglia B, Fabbri R, Paradisi R, Venturoli S, Battaglia C. Cigarette smoking and cardiovascular risk in young women with polycystic ovary syndrome. International journal of fertility & sterility. 2014;7(4):301-12. Epub 2014/02/13. PubMed PMID: 24520500; PubMed Central PMCID: PMCPmc3901187.
13. Aktaran Ş, Akarsu E, Çelik A, Altunören O. Correlation of elevated plasma homocysteine levels with insulin resistance regardless of obesity in polycystic ovary syndrome. Turkiye Klinikleri Journal of Medical Sciences. 2007;27(4):508-12.
14. Loverro G, Lorusso F, Mei L, Depalo R, Cormio G, Selvaggi L. The plasma homocysteine levels are increased in polycystic ovary syndrome. Gynecologic and obstetric investigation. 2002;53(3):157-62. Epub 2002/06/08. doi: 58367. PubMed PMID: 12053100.
15. Eskandari Z, Sadrkhanloo RA, Nejati V, Razi M, Tizroo GR. Independent to glucose and E2 the PCOS women show significantly higher hemocysteine level versus non PCOS. International Journal of Fertility and Sterility. 2012;6:107-8.
16. Celik C, Bastu E, Abali R, Alpsoy S, Guzel EC, Yeh J. The relationship between copper, homocysteine and early vascular disease in non-obese women with polycystic ovary syndrome. Fertility and sterility. 2012;98(3):S211.
17. Atanasova Boshku A, Georgievski O. Homocysteine as a marker for cardiovascular risk assessment in women with polycystic ovary syndrome. Biochimica Clinica. 2013;37:S509.
18. Pourteymour Fard Tabrizi F, Mehrzad Sadaghiani M. Relationships between total homocysteine level and anthropometric variables in infertile women with polycystic ovary syndrome. Iranian journal of reproductive medicine. 2013;11:118-9.
19. Chakravarty BN, Chakraborty P, Ghosh A. Hyperhomocysteinemia rather than hyperinsulinemia-the major determinant for long-term thrombophilic manifestations of polycystic ovary syndrome. Human Reproduction. 2014;29:i75.

20. Tsanadis G, Vartholomatos G, Korkontzelos I, Avgoustatos F, Kakosimos G, Sotiriadis A, et al. Polycystic ovarian syndrome and thrombophilia. Human reproduction (Oxford, England). 2002;17(2):314-9. Epub 2002/02/01. PubMed PMID: 11821270.
21. Luque-Ramírez M, Mendieta-Azcona C, del Rey Sánchez JM, Matíes M, Escoba-Morreale HF. Effects of an antiandrogenic oral contraceptive pill compared with metformin on blood coagulation tests and endothelial function in women with the polycystic ovary syndrome: Influence of obesity and smoking. European Journal of Endocrinology. 2009;160(3):469-80.
22. Kaya C, Pabuccu R, Berker B, Satiroglu H. Plasma interleukin-18 levels are increased in the polycystic ovary syndrome: relationship of carotid intima-media wall thickness and cardiovascular risk factors. Fertility and sterility. 2010;93(4):1200-7. Epub 2009/01/10. doi: 10.1016/j.fertnstert.2008.10.070. PubMed PMID: 19131059.
23. Glueck CJ, Iyengar S, Goldenberg N, Smith LS, Wang P. Idiopathic intracranial hypertension: associations with coagulation disorders and polycystic-ovary syndrome. The Journal of laboratory and clinical medicine. 2003;142(1):35-45. Epub 2003/07/25. doi: 10.1016/s0022-2143(03)00069-6. PubMed PMID: 12878984.
24. Glueck CJ, Wang P, Bornovali S, Goldenberg N, Sieve L. Polycystic ovary syndrome, the G1691A factor V Leiden mutation, and plasminogen activator inhibitor activity: associations with recurrent pregnancy loss. Metabolism: clinical and experimental. 2003;52(12):1627-32. Epub 2003/12/12. PubMed PMID: 14669168.
25. Moini A, Tadayon S, Tehranian A, Yeganeh LM, Akhoond MR, Yazdi RS. Association of thrombophilia and polycystic ovarian syndrome in women with history of recurrent pregnancy loss. Gynecological endocrinology : the official journal of the International Society of Gynecological Endocrinology. 2012;28(8):590-3. Epub 2012/03/29. doi: 10.3109/09513590.2011.650754. PubMed PMID: 22452370.
26. Schachter M, Raziel A, Friedler S, Strassburger D, Bern O, Ron-El R. Insulin resistance in patients with polcystic ovary syndrome is associated with elevated plasma homocysteine. Human Reproduction. 2003;18(4):721-7.
27. Chakraborty P, Goswami SK, Rajani S, Sharma S, Kabir SN, Chakravarty B, et al. Recurrent pregnancy loss in polycystic ovary syndrome: role of hyperhomocysteinemia and insulin resistance. PloS one. 2013;8(5):e64446. Epub 2013/05/24. doi: 10.1371/journal.pone.0064446. PubMed PMID: 23700477; PubMed Central PMCID: PMCPmc3660299.
28. Vrbikova J, Tallova J, Bicikova M, Dvorakova K, Hill M, Starka L. Plasma thiols and androgen levels in polycystic ovary syndrome. Clinical chemistry and laboratory medicine : CCLM / FESCC. 2003;41(2):216-21. Epub 2003/04/02. doi: 10.1515/cclm.2003.035. PubMed PMID: 12667010.
29. Badawy A, State O, El Gawad SSA, El Aziz OA. Plasma homocysteine and polycystic ovary syndrome: The missed link. European Journal of Obstetrics Gynecology and Reproductive Biology. 2007;131(1):68-72.
30. Guler I, Himmetoglu O, Turp A, Erdem A, Erdem M, Onan MA, et al. Zinc and homocysteine levels in polycystic ovarian syndrome patients with insulin resistance. Biological Trace Element Research. 2014;158(3):297-304.
31. Tsouma I, Kouskouni E, Gennimata V, Demeridou S, Boutsikou M, Grigoriou V, et al. Leptin levels in women with polycystic ovaries undergoing ovarian stimulation: relation to lipoprotein profiles. In vivo (Athens, Greece). 2014;28(5):989-92. Epub 2014/09/06. PubMed PMID: 25189919.
32. Pedroso DCC, Miranda-Furtado CL, Kogure GS, Meola J, Okuka M, Silva C, et al. Inflammatory biomarkers and telomere length in women with polycystic ovary syndrome. Fertility and sterility.

2015;103(2):542-7.e2.
33. Carmina E, Chu MC, Longo RA, Rini GB, Lobo RA. Phenotypic variation in hyperandrogenic women influences the findings of abnormal metabolic and cardiovascular risk parameters. The Journal of clinical endocrinology and metabolism. 2005;90(5):2545-9. Epub 2005/02/25. doi: 10.1210/jc.2004-2279. PubMed PMID: 15728203.
34. Kucuk M, Kilic-Okman T. Hormone profiles and clinical outcome after laparoscopic ovarian drilling in women with polycystic ovary syndrome. Medical science monitor : international medical journal of experimental and clinical research. 2005;11(1):Cr29-34. Epub 2004/12/23. PubMed PMID: 15614192.
35. Yilmaz N, Pektas M, Tonguc E, Kilic S, Gulerman C, Gungor T, et al. The correlation of plasma homocysteine with insulin resistance in polycystic ovary syndrome. The journal of obstetrics and gynaecology research. 2008;34(3):384-91. Epub 2008/08/09. PubMed PMID: 18686355.
36. Kumar PS, Ananthanarayanan PH, Rajendiran S. Cardiovascular risk markers and thyroid status in young Indian women with polycystic ovarian syndrome: A case-control study. Journal of Obstetrics and Gynaecology Research. 2014;40(5):1361-7.
37. Wijeyaratne CN, Nirantharakumar K, Balen AH, Barth JH, Sheriff R, Belchetz PE. Plasma homocysteine in polycystic ovary syndrome: does it correlate with insulin resistance and ethnicity? Clinical endocrinology. 2004;60(5):560-7. Epub 2004/04/24. doi: 10.1111/j.1365-2265.2004.02019.x. PubMed PMID: 15104558.
38. Espinós-Gómez JJ, Rodriguez-Espinosa J, Ordóñez-Llanos J, Calaf-Alsina J. Metabolic syndrome in Mediterranean women with polycystic ovary syndrome: when and how to predict its onset. Gynecological Endocrinology. 2012;28(4):264-8.
39. Mohan S, Priya V. Lipid peroxidation, glutathione, ascorbic acid, vitamin E, antioxidant enzyme and serum homocysteine status in patients with polycystic ovary syndrome. Biology and Medicine. 2009;1(3):44-9.
40. Morgante G, La Marca A, Setacci F, Setacci C, Petraglia F, De Leo V. The cardiovascular risk factor homocysteine is not elevated in young women with hyperandrogenism or hypoestrogenism. Gynecologic and obstetric investigation. 2002;53(4):200-3. Epub 2002/08/21. PubMed PMID: 12186983.
41. Kaya C, Cengiz SD, Berker B, Demirtaş S, Cesur M, Erdoğan G. Comparative effects of atorvastatin and simvastatin on the plasma total homocysteine levels in women with polycystic ovary syndrome: a prospective randomized study. Fertility and sterility. 2009;92(2):635-42.
42. Kaya C, Erkan AF, Cengiz SD, Dünder I, Demirel OE, Bilgihan A. Advanced oxidation protein products are increased in women with polycystic ovary syndrome: relationship with traditional and nontraditional cardiovascular risk factors in patients with polycystic ovary syndrome. Fertility and sterility. 2009;92(4):1372-7.
43. Yilmaz M, Biri A, Bukan N, Karakoç A, Sancak B, Törüner F, et al. Levels of lipoprotein and homocysteine in non-obese and obese patients with polycystic ovary syndrome. Gynecological Endocrinology. 2005;20(5):258-63.
44. Palep-Singh M, Picton HM, Yates ZR, Barth JH, Balen AH. Plasma homocysteine concentrations and the single nucleotide polymorphisms in the methionine synthase gene (MTR 2756A>G): Associations with the polycystic ovary syndrome An observational study. European journal of obstetrics, gynecology, and reproductive biology. 2008;138(2):180-6. Epub 2008/02/19. doi: 10.1016/j.ejogrb.2007.12.015. PubMed PMID: 18281142.
45. Erdoğan M, Karadeniz M, Alper GE, Tamsel S, Uluer H, Çağlayan O, et al. Thrombin-activatable fibrinolysis inhibitor and cardiovascular risk factors in polycystic ovary syndrome. Experimental and

Clinical Endocrinology and Diabetes. 2008;116(3):143-7.
46. Davari-Tanha F, Hemati T, Moghadami-Tabrizi N, Salmanian B, Javadian P. High plasma homocysteine and insulin resistance in patients with polycystic ovarian syndrome. Iranian journal of reproductive medicine. 2011;9:41-2.
47. Karadeniz M, Erdoan M, Ayhan Z, Yalcn M, Olukman M, Cetinkalp S, et al. Effect of G2706A and G1051A polymorphisms of the ABCA1 gene on the lipid, oxidative stress and homocystein levels in Turkish patients with polycystc ovary syndrome. Lipids in health and disease. 2011;10.
48. Atanasova Boshku A, Ivanova Panova D, Biljali S. Plasma homocysteine levels in woman with polycystic ovary syndrome in R.Macedonia. Clinical Chemistry and Laboratory Medicine. 2014;52:S251.
49. Atanasova Boshku AM, Ivanova Panova D. Homocysteine as a marker for inflammation in woman with polycystic ovary syndrome. FEBS Journal. 2014;281:128-9.
50. Pamuk BO, Torun AN, Kulaksizoglu M, Ertugrul D, Ciftci O, Kulaksizoglu S, et al. Asymmetric dimethylarginine levels and carotid intima-media thickness in obese patients with polycystic ovary syndrome and their relationship to metabolic parameters. Fertility and sterility. 2010;93(4):1227-33. Epub 2008/12/26. doi: 10.1016/j.fertnstert.2008.10.073. PubMed PMID: 19108830.
51. Boulman N, Levy Y, Leiba R, Shachar S, Linn R, Zinder O, et al. Increased C-reactive protein levels in the polycystic ovary syndrome: a marker of cardiovascular disease. The Journal of clinical endocrinology and metabolism. 2004;89(5):2160-5. Epub 2004/05/06. doi: 10.1210/jc.2003-031096. PubMed PMID: 15126536.
52. Markou A, Androulakis II, Mourmouris C, Tsikkini A, Samara C, Sougioultzis S, et al. Hepatic steatosis in young lean insulin resistant women with polycystic ovary syndrome. Fertility and sterility. 2010;93(4):1220-6.
